# Scrotal Migration of the Ventriculoperitoneal Shunt: A Case Report and Review of the Literature

**DOI:** 10.7759/cureus.63384

**Published:** 2024-06-28

**Authors:** Farrukh Javeed, Maryam Tariq, Hiba Butt, Lal Rehman

**Affiliations:** 1 Neurosurgery, Jinnah Postgraduate Medical Centre, Karachi, PAK; 2 Neurosurgery, Karachi Medical and Dental College, Karachi, PAK

**Keywords:** hydrocephalus, scrotal migration, shunt complications, shunt migration, ventriculoperitoneal shunt

## Abstract

Ventriculoperitoneal (VP) shunt placement is the most frequently used treatment for hydrocephalus. This procedure is not always free of complications, and patients may need additional surgeries to overcome these complications. We are presenting the case of a seven-month-old baby who underwent myelomeningocele repair and VP shunt placement 13 days ago and now presents with inguinal swelling extending into the scrotum. The radiological workup revealed that the peritoneal end of the VP shunt had migrated to the scrotum, causing hydrocele. The shunt was relocated to the abdomen after a right herniotomy and sac reduction. He was discharged on the second postoperative day without any complications, and the further recovery was good at three months. Scrotal migration of a VP shunt is a rare complication and can be avoided by careful early assessment of inguinal hernia or patent processus vaginalis and its surgical repair.

## Introduction

A ventriculoperitoneal (VP) shunt is a common surgical method employed to direct the flow of cerebrospinal fluid (CSF) towards the peritoneum and thereby treat the hydrocephalus. Despite significantly reducing morbidity and mortality in hydrocephalus patients, VP shunts often cause further complications that require surgical intervention [1]. Infection, blockage, and leakage are some of the complications, the most common being shunt dysfunction [2]. A rare complication is shunt migration, in which a proximal or distal catheter displaces from its original location and results in the failure of the procedure [3]. We aim to document one of the rare cases of shunt complications and their management. 

## Case presentation

**Figure 1:**
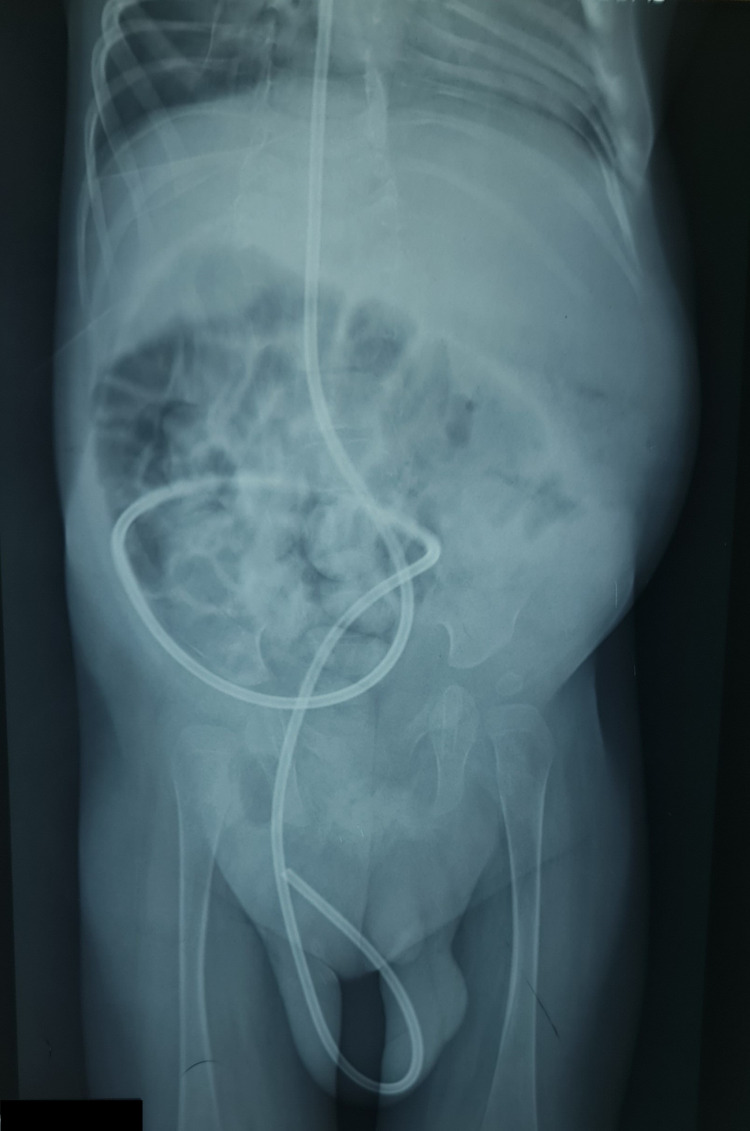
X-ray showing the lower end of the VP shunt passing into the right scrotum through the inguinal canal.

A seven-month-old male child presented with increased head circumference, irritability, and a few episodes of vomiting and swelling at the right inguinal region extending into the scrotum for the last five days. He was operated on 13 days ago with myelomeningocele repair and placement of a right-sided VP shunt through Keen’s point for congenital hydrocephalus. Examination on re-admission showed hydrocele and a palpable lower end of the VP shunt in the scrotum. Initially, X-ray shunt series were done, and the shunt tube was identified intact without any breakage. The lower end of the VP shunt was seen passing into the inguinal area and then into the right scrotum (Figure 1).

**Figure 2:**
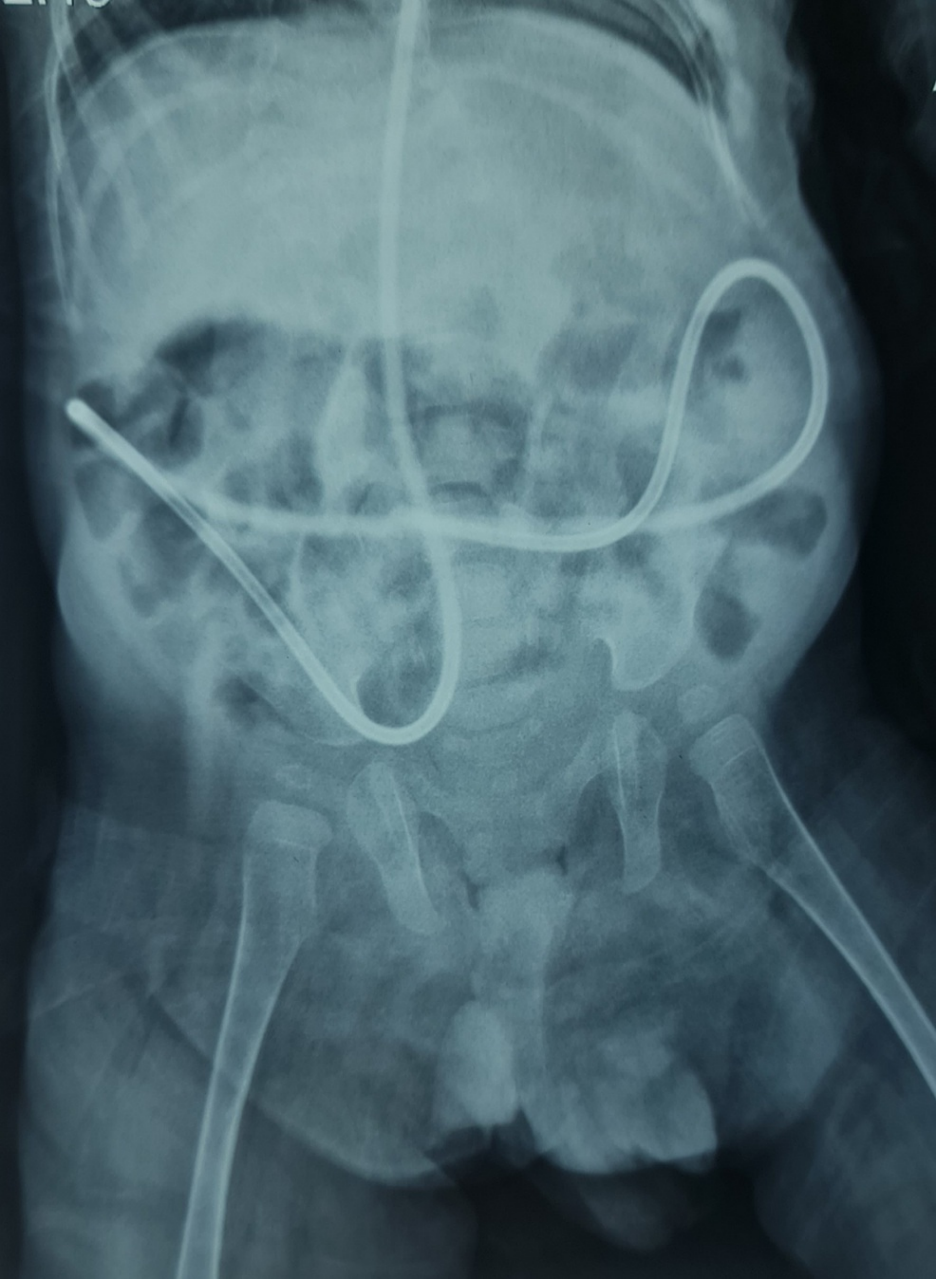
X-ray abdomen showing VP shunt successfully reduced back into the peritoneum.

With the suspicion of shunt migration into the scrotum, the pediatric surgery team was taken on board, and surgical intervention was planned to reduce the shunt back into the peritoneum. A right herniotomy was performed under general anesthesia to reduce the VP shunt back into the peritoneum. A hernia sac containing the lower end of the VP shunt and a loop of bowel was identified during the procedure. The vas deferens and vessels were preserved. Postoperatively, the patient was managed with intravenous antibiotics and analgesics. He was allowed oral intake on the same day of the procedure, which was well tolerated. An X-ray of the abdomen done postoperatively showed the lower end of the VP shunt was successfully reduced in the peritoneum (Figure 2).

The child was discharged on the second postoperative day. The child was called for follow-up in the next week. No further complications were identified over the next three months.

## Discussion

Ventriculoperitoneal shunt surgery can present with many complications, including obstruction, seizures, disconnection, infection, over-drainage, and abdominal organ perforation. Scrotal migration is one of the rare mechanical complications of shunt surgery, with around 50 cases reported worldwide [4]. It is unclear why this occurs more frequently in children; however, it may be related to the increased prevalence of unobliterated processus vaginalis and lower peritoneal volume leading to scrotal migration [5].

The earliest case report on migration of a ventriculoperitoneal shunt was by P. S. Ramani in 1974 [6]. There were a few other case reports in the same decade by Grosfeld JL and Bristow [7,8]. They also gave their theories for this VP shunt migration. Ramani suggested that the unobliterated processus vaginalis might be the causative factor. The case series by Grosfeld et al. suggests that this shunt migration is due to increased intra-abdominal pressure secondary to an inadequate peritoneal absorption rate. The case reported by Kimura et al. was an adult male patient, and he gave the opinion that the catheter slipped from the subarachnoid space through the thin patent vaginal process, causing local inflammation and leading to occlusion of the path [9]. A recent study also reported similar findings [10].

Distal catheter migration through the processus vaginalis into the scrotum is extremely rare [11]. Studies demonstrate that 16.8% of the pediatric population presents with inguinal hernias following shunt placement surgery, mainly attributed to patent processus vaginalis, which is common in 90% of male newborns and 15% of adult males [12].

Reports suggest that factors such as inadequate peritoneal cavity absorbing ability, bowel peristalsis exerting pressure on the catheter, muscular weaknesses related to meningomyelocele, and the closure of a large meningomyelocele can all lead to an increase in intra-abdominal pressure, leading to the migration of the VP shunt into the scrotum [11,13]. VP shunt migration usually occurs on the right side; only a few cases have been reported on the left side, which was also the case in our patient [14,15,16].

Our patient is a male child with a patent processus vaginalis, either congenital or because of peritoneal CSF drainage. Scrotal migration of the VP shunt can cause secondary hydrocele formation and shunt malfunction, leading to worsening of the hydrocephalus.

## Conclusions

The placement of a VP shunt is commonly employed to redirect the flow of CSF. The patent processus vaginalis and increased intra-abdominal pressure are unavoidable factors responsible for shunt migration. It may also result from the detachment of a ligature or a loose ligature stitch. Early detection of patent processus vaginalis and surgical closure may prevent such a complication in selected cases. 
